# Seasonal trophic controls drive population variability in a foundational marine copepod

**DOI:** 10.1038/s41598-025-19919-2

**Published:** 2025-10-15

**Authors:** Isabel A. Honda, Lucas P. Medeiros, Cameron R. S. Thompson, Gregory L. Britten, Jeffrey A. Runge, Rubao Ji

**Affiliations:** 1 Biology Department, Woods Hole Oceanographic Institution, Woods Hole, MA USA; 2 Department of Civil and Environmental Engineering, Massachusetts Institute of Technology, Cambridge, MA USA; 3 Northeastern Regional Association of Coastal Ocean Observing Systems (NERACOOS), Portsmouth, USA; 4 Department of Earth, Atmospheric, and Planetary Sciences, Massachusetts Institute of Technology, Cambridge, MA USA; 5 Darling Marine Center School of Marine Sciences, University of Maine, Walpole, ME USA; 6 Present Address: Carbon Technology Research Foundation, Oxford, UK

**Keywords:** *Calanus finmarchicus*, Gulf of Maine, Seasonal trophic interactions, Convergent cross mapping, Population variability, Climate-change ecology, Community ecology, Ecological modelling, Ecological networks, Ecosystem ecology, Population dynamics, Biooceanography, Ocean sciences, Marine biology

## Abstract

Understanding the trophic drivers of zooplankton population variability is critical for predicting ecosystem responses to climate change. In the Gulf of Maine, the copepod *Calanus finmarchicus* is a foundational species linking primary producers to higher trophic levels, yet the biotic drivers shaping its seasonal and interannual abundances remain incompletely understood. Here, we assess how predators impact *C. finmarchicus* abundances using over four decades of survey data. We find strong evidence for seasonally-structured trophic control, with spring *C. finmarchicus* abundances driving mid-year predator increases, which subsequently imposes significant top-down pressure on fall *C. finmarchicus* populations. This interplay is especially pronounced in the deep, retentive inner basins of the Gulf of Maine, where predator-prey dynamics tend to dominate over advective exchange. Our results reveal shifting interactions between bottom-up and top-down controls, highlighting the need to incorporate seasonal trophic mechanisms into ecosystem models to improve projections under further environmental change.

**Supplementary Information:**

The online version contains supplementary material available at 10.1038/s41598-025-19919-2.

## Introduction

Disentangling predator-prey relationships in ecosystems has been a major challenge in ecology for more than a century. A complex interplay of both intrinsic and extrinsic nonlinear dynamics and environmental factors obscure direct causal links between species, making detecting trophic controls particularly difficult^1^. A foundational framework for modeling predator-prey interactions was established more than 100 years ago by Alfred J. Lotka and Vito Volterra, who independently developed a set of nonlinear differential equations to describe population cycles driven by density-dependent feedbacks in which prey populations grow exponentially without predators, and predator abundances decrease in the absence of sufficient prey^2,3^. Such dynamics have been exemplified across terrestrial ecosystems, including the classic case of the snowshoe hares and lynxes in Canada, where historical fur-trapping records revealed periodic oscillations in their populations^4,5^. In marine ecosystems, similar patterns have been observed in the Adriatic Sea during World War I, as Volterra’s application of the model could effectively explain the proliferation of sharks as result of reduced fishing pressure^3^. Hence, the original Lotka-Volterra models have been useful for capturing idealized population cycles and have since been extended to incorporate more complex biological behaviors^6–8^. However, many population models rely on simplifying assumptions, such as spatial homogeneity, constant interaction strengths, no temporal lags, and static long-term dynamics, which are not always valid generalizations in complex ecological systems.

To address these limitations in traditional models, ecologists and mathematicians have developed a wide array of mechanistic models that incorporate more realistic features into population dynamics. Yet, two major challenges permeate throughout mathematical ecology and population dynamics modeling. First, since most state variables in a system are unobserved, we need approaches that can reconstruct useful information using only the few variables that we have data for. Second, the precise form of the mechanistic governing equations is usually uncertain, so flexible, nonparametric methods are necessary to capture nonlinear population dynamics. Empirical dynamic modeling (EDM) has emerged as a particularly useful approach to overcome these challenges by combining nonparametric function approximation with state-space reconstruction to understand and forecast complex, nonlinear systems^9^. In particular, convergent cross mapping (CCM) is a technique within this framework that enables the detection of dynamic relationships in partially observed dynamical systems without assuming fixed interaction structures^10^. CCM has gained traction in recent years due to its success in uncovering potentially causal relationships in a wide range of ecosystems, including terrestrial environments, freshwater lakes and rivers, and marine food webs^11–14^.

**Fig. 1:**
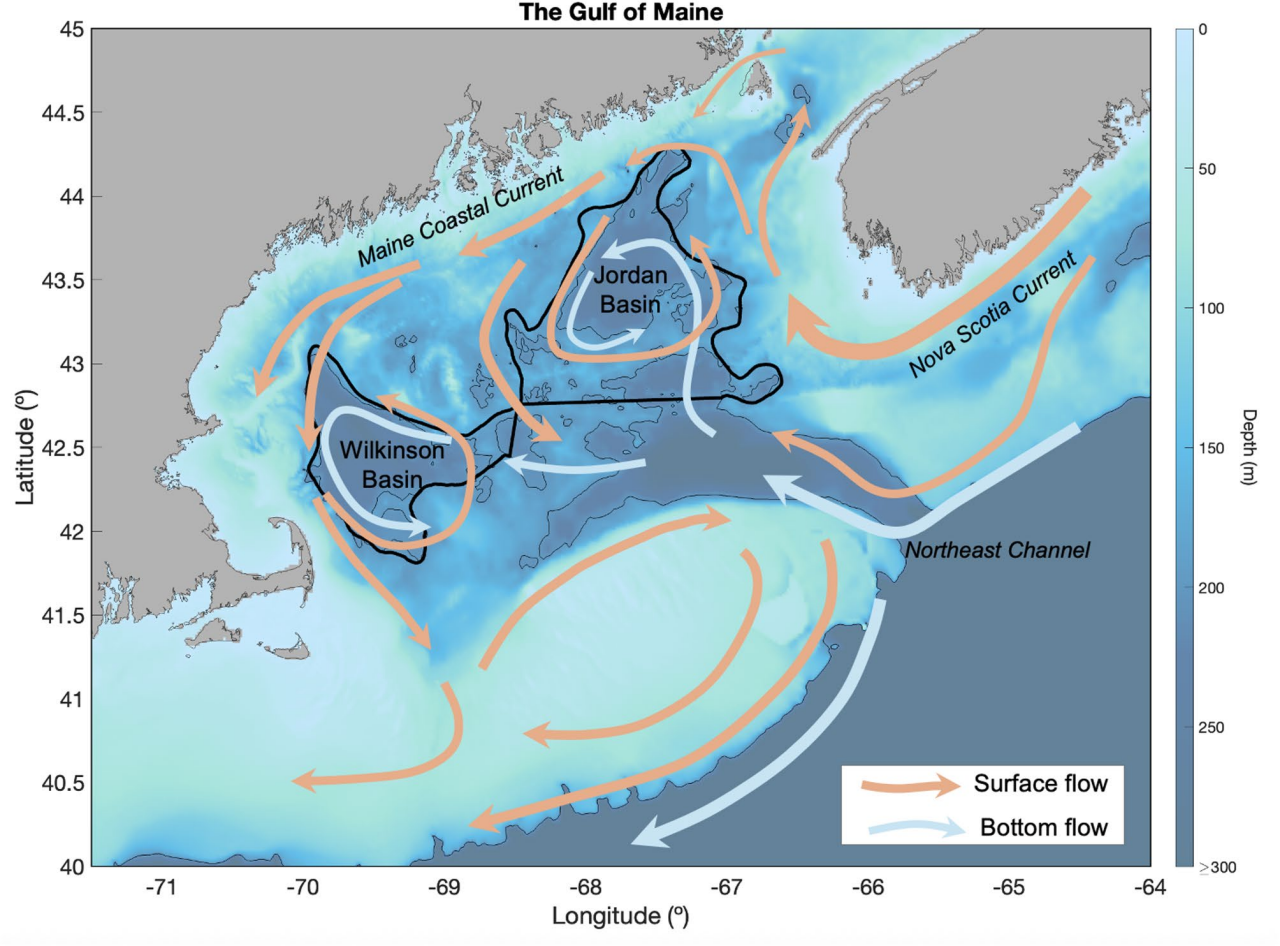
Bathymetry of the Gulf of Maine with spatial areas indicating Wilkinson Basin and Jordan Basin. Directionality of surface and bottom flow are denoted by orange and and blue arrows (respectively).

The Gulf of Maine is an ecologically and economically important marine ecosystem, supporting a diverse community of zooplankton, forage fish, and higher trophic level predators^15^. Wilkinson and Jordan Basins form the deep regions of the inner Gulf of Maine (Fig. 1), and the Gulf’s unique circulation structure leads to long residence times in these basins^16–18^. This makes the inner Gulf of Maine a valuable refuge due to the retention of nutrients, plankton, and overwintering populations of key species^18,19^. Within this complex ecosystem, the copepod *Calanus finmarchicus* plays a foundational role, as it acts as a bridge between primary producers and higher trophic level consumers^20^. However, despite its critical position in the food web, the drivers of *C. finmarchicus* abundance remain incompletely understood, as it is extremely difficult to differentiate the multiple mechanisms affecting population growth and mortality^21,22^. Additionally, the complex interplay between internal production versus external exchange of *C. finmarchicus* populations in Wilkinson and Jordan Basins has been a major focus of study in recent years, particularly as climate change threatens to disrupt regional circulation patterns and alter the phenology of biological processes that sustain these populations^15,17,23–25^. Since the Gulf of Maine is an intensively sampled marine ecosystem, there is a unique opportunity to integrate this high-resolution, multi-decadal plankton survey data with modern statistical techniques to disentangle the relative roles of local production, predation, and advective exchange driving *C. finmarchicus* population dynamics^25,26^.

Here, we apply CCM along with other statistical approaches to investigate the predator-prey relationships influencing populations of *C. finmarchicus* in the inner basins of the Gulf of Maine. Past studies indicate that temperature and advection likely are not the primary drivers of population variability, so we hypothesize that internal production driven by seasonal density-dependent predator-prey interactions play a key role in regulating the population dynamics of *C. finmarchicus* in these basins^17,23,24^. Our analysis extends beyond previous CCM studies focused on marine ecosystems (e.g.^27–29^), as we resolve season-specific trophic links to quantify the distinct contributions of multiple predators’ impact on *C. finmarchicus* dynamics across the spring, summer, and autumn. We apply these rigorous statistical techniques in a novel, seasonally-partitioned framework, offering a transferable template for other ecosystems to test classical predator-prey theory under real-world complexity. Our results indicate that siphonophores, euphausiids, and chaetognaths exert strong, seasonally-resolved top-down control on *C. finmarchicus*. These findings provide new insight and statistical evidence for the seasonal biotic mechanisms driving this foundational marine copepod in the Gulf of Maine.

## Results

To assess the role of predation in influencing *C. finmarchicus* population dynamics, we utilize a season-specific approach that combines correlation analyses, boosted regression trees (BRTs), and CCM. First, we analyze correlations and dynamic coupling between spring and fall abundances of *C. finmarchicus* to understand whether the spring condition of one generation of *C. finmarchicus* influences the abundances of the following generations in the fall, as *C. finmarchicus* can undergo multiple generations per year in the Gulf of Maine^30,31^. We then apply BRTs to rank key predators according to their predictive influence on both spring and fall *C. finmarchicus* abundances, which gives insights into which predators are more likely to be bottom-up driven, and which primarily control the population of *C. finmarchicus* through top-down control. Finally, we use Spearman rank correlations and CCM to test for dynamic coupling between spring and fall *C. finmarchicus* abundances and spring-summer abundances of each hypothesized predator to assess the relative role of bottom-up/top-down controls and how these biotic interactions may change throughout the year in the inner basins of the Gulf of Maine.

### Spring vs. fall abundance

**Fig. 2:**
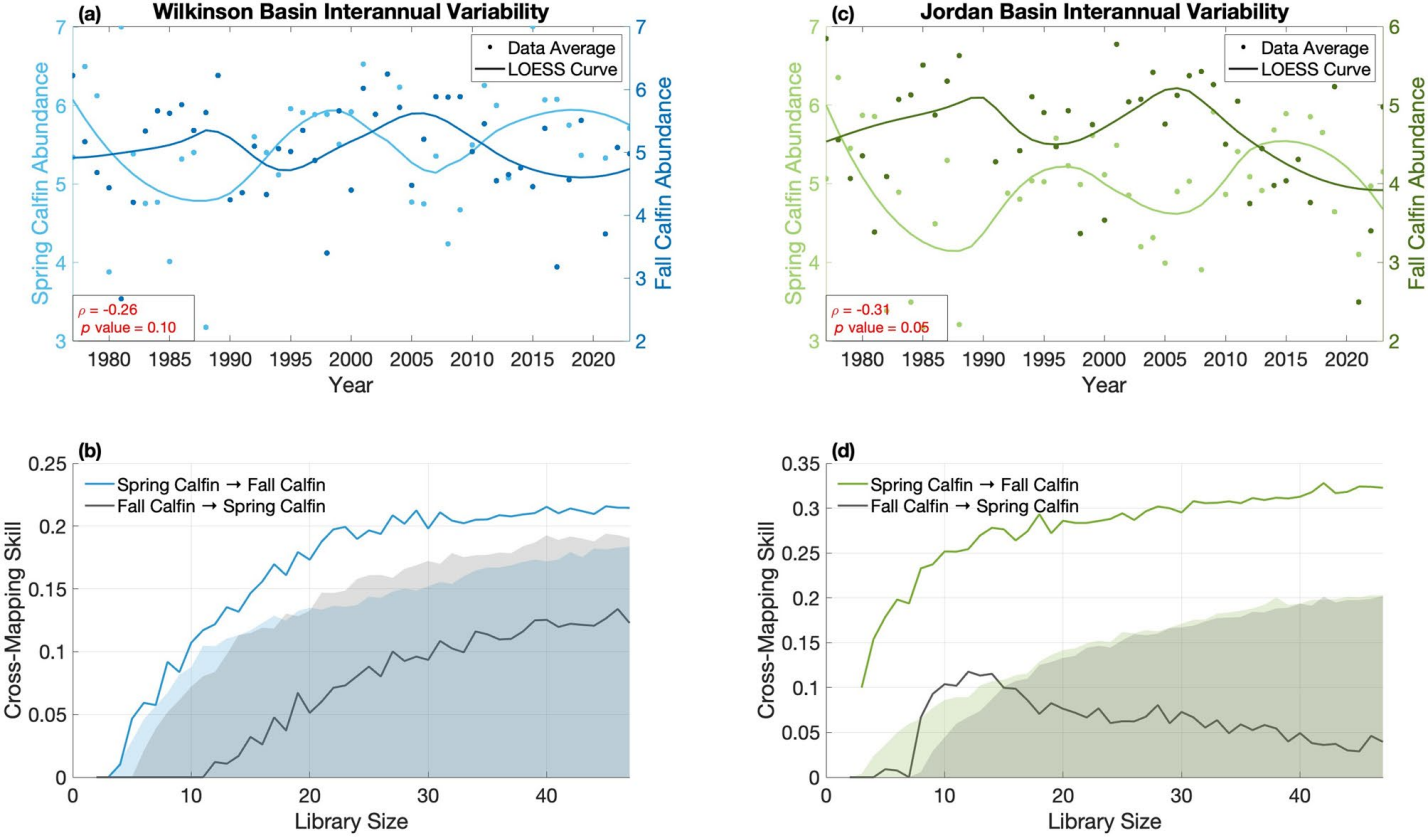
Mean spring and fall interannual trends of *C. finmarchicus* (Calfin) in Wilkinson Basin (**a**) and Jordan Basin (**c**), with dots representing the average seasonal abundance (units of ln[abundance m^− 3^ + 1]) and lines representing the LOESS smooth curve for easier visualization of trends. CCM results for Wilkinson Basin (**b**) and Jordan Basin (**d**) indicate significant evidence for Spring Calfin → Fall Calfin causality.

There is a negative correlation between the average spring and fall abundances of *C. finmarchicus* in both Wilkinson Basin (ρ = -0.26, *p* = 0.10, Fig. 2a) and Jordan Basin (ρ = -0.31, *p* = 0.05, Fig. 2c). CCM results indicate that there is significant evidence for dynamic coupling (i.e., that spring abundances influence fall abundances within the same year), as the cross-mapping skill increases with library size and is greater than the blue and green shaded 95% confidence region given by the null distribution (Fig. 2b & d). The magnitude of the cross-mapping skill quantifies the strength of the dynamic coupling effect, with higher values indicating stronger linkage^10^. For spring → fall *C. finmarchicus* dynamics, this effect is greater in Jordan Basin (converges to a cross-mapping skill of 0.32; Fig. 2d) than in Wilkinson Basin (converges to a cross-mapping skill of 0.21; Fig. 2b). However, there is insufficient evidence to support dynamic coupling in the opposite direction (i.e., fall abundances influence spring abundances in the same year), as grey lines do not fall above the grey shaded region. These results agree with intuition and the hypothesis that the spring condition of *C. finmarchicus* can result in a cascading chain of events to drive either high or low fall abundances within the same year, but not vice versa.

### Bottom-up and top-down BRT assessment

**Fig. 3:**
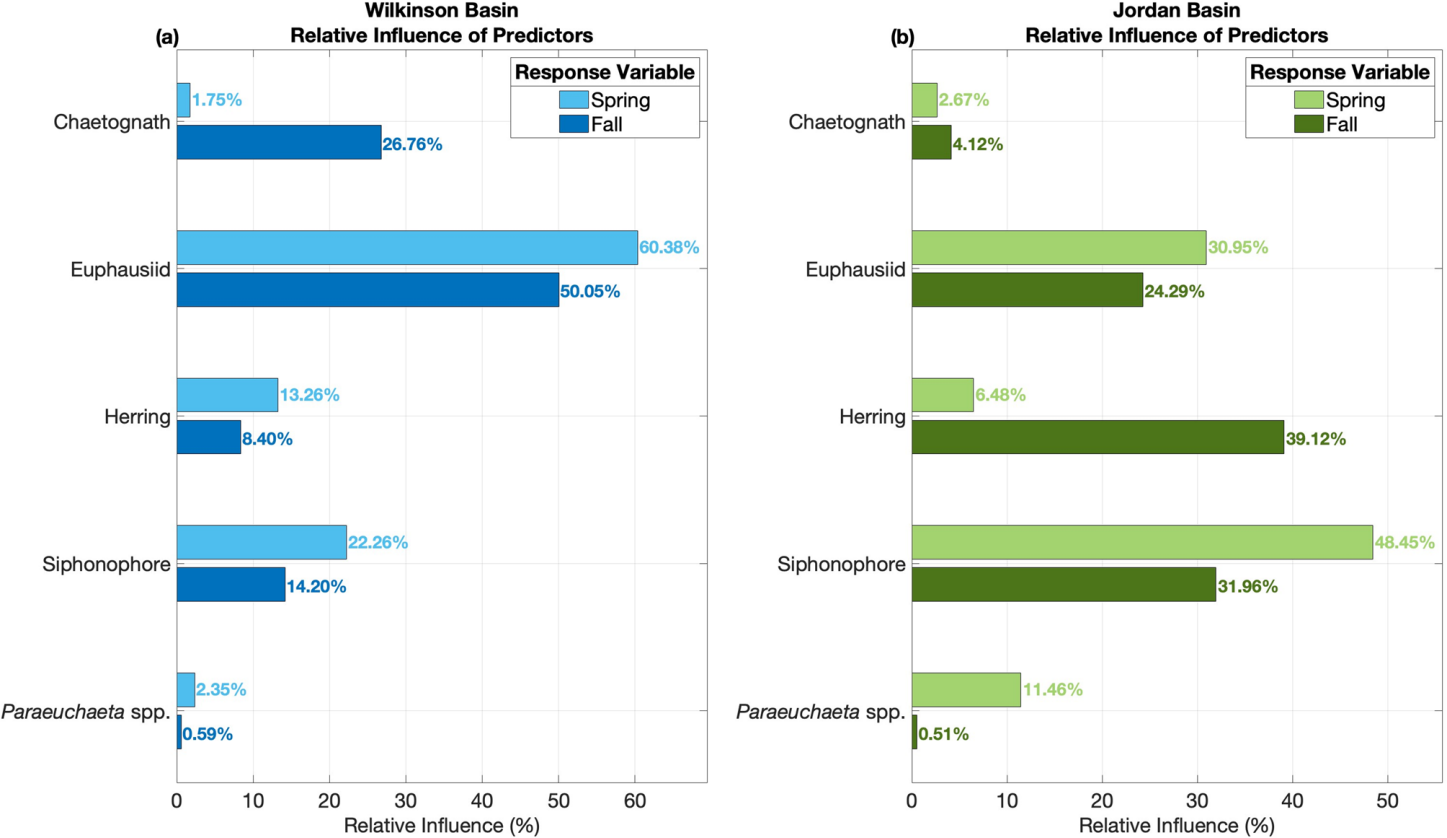
Boosted regression tree results for Wilkinson Basin (**a**) and Jordan Basin (**b**). Bars give each predator’s relative predictive influence (%), calculated as the normalized frequency at which each predator was selected as improving model fit. Results show spring-summer predator averages being used to predict *C. finmarchicus* (Calfin) abundance in spring (light blue/green) and fall (dark blue/green). Here, the Spring response variable indicates bottom-up control (Spring Calfin → Predators) while the Fall response variable indicates top-down control (Predators → Fall Calfin).

We used BRTs for populations in Wilkinson (Fig. 3a) and Jordan Basin (Fig. 3b) to assess how effectively spring-summer abundances of different predators predict spring and fall *C. finmarchicus* abundances. Using a spring *C. finmarchicus* response variable with the BRT represents bottom-up control (i.e., spring *C. finmarchicus* positively influencing predator abundances), while a fall *C. finmarchicus* response variable in the BRT represents top-down control (i.e., predators driving down populations of *C. finmarchicus*). Across both basins, spring-summer abundances of euphausiids (average relative influence of 41.4%), siphonophores (average relative influence of 29.2%), and herring (average relative influence of 16.8%), generally rank as having the greatest predictive influence on both spring and fall *C. finmarchicus* abundances. Partial dependence plots of the BRTs indicate that most predators exhibit a positive association with spring *C. finmarchicus* abundances and a negative association with fall abundances. Exceptions include chaetognaths in Wilkinson Basin, which are negatively associated with spring *C. finmarchicus*, and herring in Jordan Basin, which generally have a positive association with fall *C. finmarchicus* (Supplementary Information: Figures S1 and S2).

### Predator correlations & CCM

**Fig. 4:**
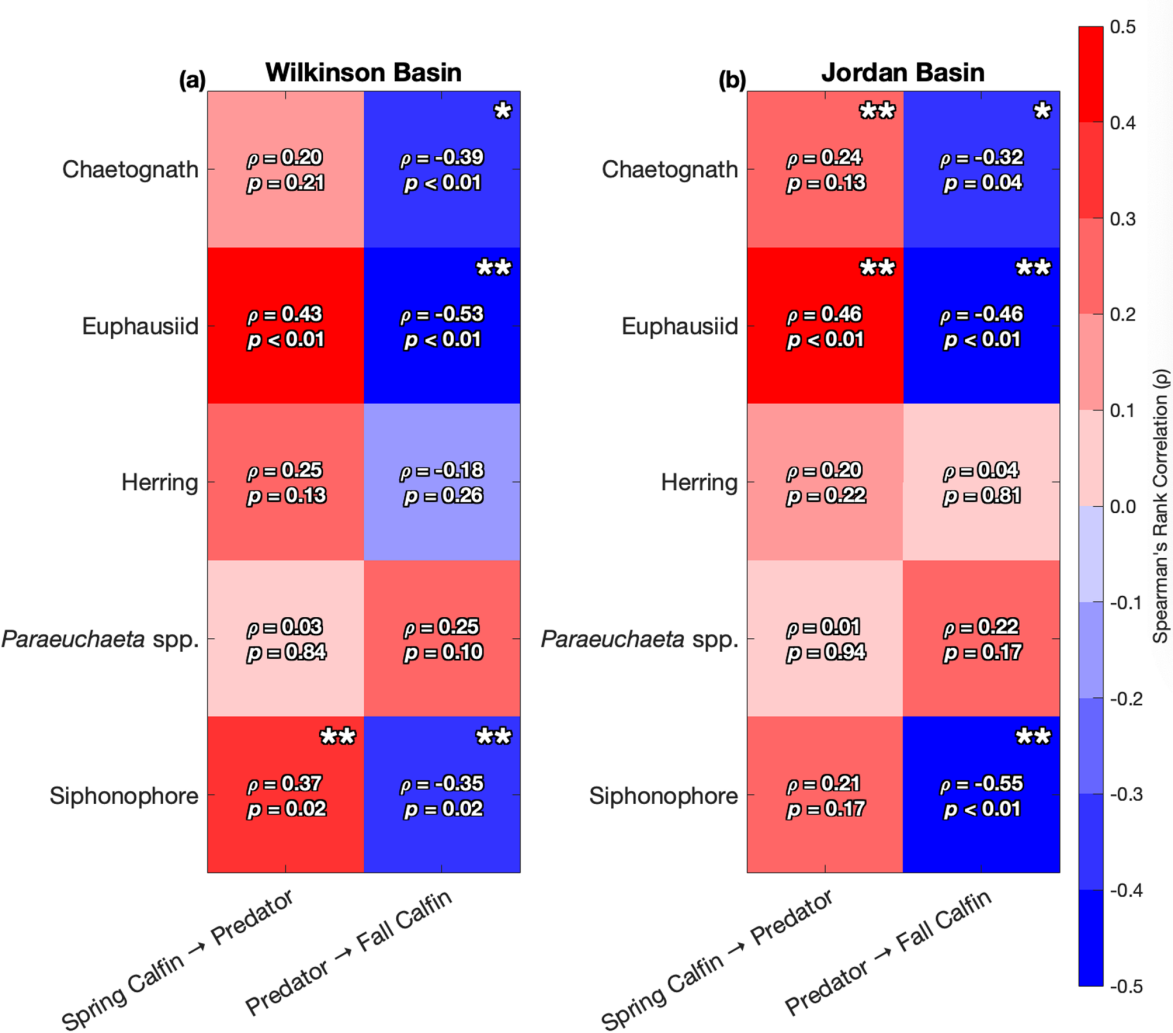
Heatmap of correlations of five potential predators of *C. finmarchicus* (Calfin) in Wilkinson Basin (**a**) and Jordan Basin (**b**). Values and colors inside the boxes indicate the Spearman’s rank correlation (ρ) and associated *p* values between spring Calfin & spring-summer predator and spring-summer predator & fall Calfin. Two asterisks (**) indicate significance of CCM to the 95% significance level, while one asterisk (*) indicates significance of CCM to the 90% significance level.

Moreover, we examined Spearman’s rank correlations (ρ), associated *p* values, and CCM significance between spring *C. finmarchicus* abundance and spring-summer predators (bottom-up control) and between spring-summer potential predators and fall *C. finmarchicus* abundance (top-down control) in Wilkinson Basin (Fig. 4a) and Jordan Basin (Fig. 4b). Positive correlations would be expected for a system dominated by bottom-up control (“Spring Calfin → Predator” column), while negative correlations would be expected for a system experiencing primarily top-down control (“Predator → Fall Calfin” column)^32,33^. Asterisks indicate the significance of CCM results for each predator and spring or fall *C. finmarchicus* abundance. As shown in Fig. 4, some highly correlated species are not significantly dynamically coupled through our CCM analysis (e.g., Spring Calfin → Euphausiids in Wilkinson Basin), while some significant CCM relationships are not significantly correlated (e.g., Spring Calfin → Chaetognaths in Jordan Basin). In Wilkinson Basin, spring-summer abundances of euphausiids and siphonophores have significant correlations (*p* value ≤ 0.05) with both spring and fall *C. finmarchicus* abundances. However, based on the CCM results, only siphonophores are dynamically coupled to *C. finmarchicus* from the bottom-up perspective, while chaetognaths, euphausiids, and siphonophores appear to significantly drive down fall *C. finmarchicus* abundances via top-down control. In Jordan Basin, some similar patterns emerge, except spring *C. finmarchicus* abundances appear to have a greater bottom-up influence on chaetognaths than in Wilkinson Basin. Across both basins, invertebrate predation from euphausiids and siphonophores exert the greatest top-down pressure on *C. finmarchicus* (Supplementary Information: Figures S3-S6).

## Discussion

Our findings reveal that the deep inner basins of the Gulf of Maine consistently exhibit season-specific trophic patterns characterized by spring *C. finmarchicus* abundances influencing fall abundances later in the year (Fig. 2). We hypothesized that this compensatory pattern between spring and fall abundances is driven primarily by predation. Results from correlation, BRT, and CCM analyses corroborate our theory that high spring abundances are generally conducive to high spring-summer predator abundances. We find that across both basins, invertebrate predators such as euphausiids, siphonophores, and chaetognaths particularly exhibit the strongest predictive relationships, the most significant correlations, and clearest dynamic linkages (Figs. 3 and 4). Our results further indicate that these predators are exerting top-down pressure on *C. finmarchicus*, consequently driving low fall *C. finmarchicus* abundances in the same year (Figs. 3 and 4).

Observed seasonal interactions in the basins of the Gulf of Maine are consistent with predator-prey interactions described by more general resource-consumer dynamics. When seasonal forcing is imposed through periodic changes in resource availability or environmental conditions, predator-prey models predict alternating phases of bottom-up and top-down control that lag with the timing of those forcings. In the basins of the Gulf of Maine, spring abundance of *C. finmarchicus* is heavily influenced by the seasonal phenology of phytoplankton production^25,24^. Our statistical analyses through this study further shows a cascading chain of oscillating predator-prey interactions, where a strong spring cohort results in enhanced predation in spring and summer seasons, which consequently leads to reductions in fall *C. finmarchicus* densities.

However, the predator-prey dynamics exhibited in Wilkinson and Jordan Basins are not necessarily reflected in shallower, spatially proximate regions (e.g., Stellwagen Bank; Supplementary Information: Figure S7). Bathymetry shapes the vertical distribution of species and alters the balance between internal production and external exchange, as shallow areas are more strongly influenced by external exchange due to shorter residence times, which dilutes the coupling between predator and prey^34,35^. This results in a more complex interplay of multiple trophic interactions, which likely disrupts the formation of well-defined, anti-phase population signals between spring and fall abundances. On the other hand, the deep basins of the inner Gulf of Maine provide an important habitat for diapausing *C. finmarchicus*, as populations are able to descend more than 150 m to avoid visual predation and survive times of food scarcity in the late summer and early fall^36^. Recent work suggests that Wilkinson and Jordan Basins are dominated by internal production, with local predator-prey dynamics driving population variability more than advective inputs^23^. Here, our findings further corroborate this result, as the ability to diapause and longer residence times in these basins amplifies the compensatory signal between spring and fall population abundances, resulting in stronger, negative top-down correlations in the fall season from non-visual invertebrate predators, such as chaetognaths and siphonophores^17,37^. This small-scale spatial heterogeneity indicates that while simplified cyclical population dynamics may capture predator-prey interactions in the more isolated basins, the dynamics in shallow regions are complicated by additional ecological and physical factors. Therefore, it is important to consider a spatially explicit and mechanistically informed approach that not only considers the balance of top-down and bottom-up controls, but also how local bathymetry and the physical connectivity structure of the region impacts the relative contributions of internal versus external drivers^38,39^.

Moreover, analyzing the life history strategies and feeding modes of these potential predators can shed further insight on observed dynamic interactions. For instance, invertebrate predators such as chaetognaths, *Paraeuchaeta* spp., euphausiids, and siphonophores exhibit diverse functional traits that likely shape their interactions with *C. finmarchicus*. Chaetognaths and *Paraeuchaeta* spp. are ambush predators with short lifespans and strong diel vertical migration (DVM) behavior, which allows them to efficiently target early life stages of copepods during seasonal peaks^40,41^. Based on our CCM results, there is evidence to suggest that *C. finmarchicus* could be a significant prey for chaetognaths in Jordan Basin and chaetognaths could be driving down fall *C. finmarchicus* abundances in both basins, but patterns for *Paraeuchaeta* spp. are not clear (Fig. 4). Furthermore, siphonophores are non-visual colonial gelatinous predators that primarily reside in deeper waters and can reproduce rapidly (i.e., on the order of days to weeks) in response to prey availability, particularly in semi-enclosed basins where retention is high and flushing rates are relatively low^16,42,43^. This is similarly reflected in the strength and direction of the correlations as well as the CCM results, as our analysis indicates that *C. finmarchicus* is a significant bottom-up prey for siphonophores in Wilkinson Basin, and siphonophores exhibit significant top-down pressure on *C. finmarchicus* in both basins (Fig. 4). On the other hand, species of euphausiids may exhibit both filter-feeding and visual predation strategies^44^. Their longer life spans (one to two years) as well as their swarming migratory behavior across seasons suggest that euphausiids could have a slower yet sustained predation impact across time^45^. There is significant evidence to support that euphausiids, likely *Meganyctiphanes norvegica*^46^, exhibit top-down pressure in both basins, but the bottom-up effect from *C. finmarchicus* is only pronounced in Jordan Basin (Fig. 4). These feeding traits enable invertebrate predators to exert relatively prompt and continuous top-down pressure in the spring and summer seasons, yet it is evident that *C. finmarchicus* populations exhibit varying degrees of response to this pressure based on their population variability in the fall. Notably, from the BRT results, Jordan Basin herring appear to have relatively high predictive influence in the fall compared to other predators (Fig. 3), but exhibit weak and insignificant correlation with no evidence for dynamic coupling (Fig. 4). This could indicate that the predictive power of herring arises from indirect or context-dependent associations (such as shared environmental drivers) rather than direct dynamic coupling with fall *C. finmarchicus* abundances.

Our results further demonstrate that a season-specific and spatially explicit approach is necessary to better understand the balance between bottom-up and top-down controls in the Gulf of Maine. Previous studies have characterized this region as being predominantly bottom-up driven, influenced primarily by temperature, nutrients, and phytoplankton production^32,47^. However, our seasonally-resolved BRT and CCM analyses uncover a more nuanced picture: strong spring cohorts of *C. finmarchicus* fuel predator growth through bottom-up control, while intense predation in the late summer and fall sharply reduces overwintering *C. finmarchicus* stocks. Hence, seasonal analyses at the decadal scale are critical for analyzing population dynamics in the Gulf of Maine, as drivers of *C. finmarchicus* variability shift across the year with a complex intermingling of both bottom-up and top-down control^17,24,37^. Since *C. finmarchicus* is responsible for transferring a large fraction of primary production to higher trophic levels, we show that seasonal variability in its abundance could potentially have cascading effects both up and down the marine food chain. On the ecosystem level, such shifts could influence predator populations that are dependent on *C. finmarchicus* for food (such as the critically endangered North Atlantic Right Whale), as well as alter community competitive dynamics among zooplankton species^37,48,49^. Therefore, our findings highlight the importance of incorporating seasonal trophic complexity into ecosystem models to more accurately predict the responses of marine food webs to ongoing environmental change.

## Methods

### Data

Plankton data for this study comes from the Marine Monitoring Assessment and Prediction (MARMAP) survey (1977 to 1987) and the Ecosystem Monitoring (EcoMon) program (1988 to the present) collected by the U.S. National Oceanic and Atmospheric Administration (NOAA) Northeast Fisheries Science Center (NMFS/NEFSC). Plankton samples were collected approximately six times a year using a 61-cm bongo net with a 333-µm mesh towed at a maximum depth of 200 m at fixed or randomly selected stations. Results from a past study indicate that data collected within Wilkinson and Jordan Basins are spatially synchronized, so there is no statistically significant dependence of interannual variability on spatial location^23^. Therefore, we aggregated all data collected within the geographic boundary representing these basins (Fig. 1). For each year and individual basin, we took the mean abundance of all data collected in the spring (months April to June), the spring-summer (months April to September) and/or the fall (months October to December), generating a time series that estimates the average interannual trends across different seasons. We used spring and fall time series for *C. finmarchicus*, as these seasons are most important for its reproduction and life history. Spring corresponds to a period of rapid feeding and population growth, while fall marks when most individuals have entered diapause and are less available as prey^20^. Accordingly, we averaged predator abundances over the spring and summer months to encompass each predators’ extended and variable foraging and reproductive periods. This approach allows us to analyze both the bottom-up effect of predator responses to *C. finmarchicus* growth as well as the top-down influence of these predators on *C. finmarchicus* prior to diapause. Since *C. finmarchicus* has a wide variety of invertebrate and vertebrate predators, we tested the five most common species identified through expert opinion and literature^17,37,50,51^.

Atlantic herring data comes from the standardized spring and fall NOAA NEFSC bottom-trawl surveys spanning from 1968 to the present. Sampling stations were selected using a random stratified design. Please refer to Politis et al. (2014) for more details on survey protocols and sampling gear. Similar to the plankton data, we aggregated all bottom trawl data collected within the geographic boundary for Wilkinson and Jordan Basins to estimate average seasonal interannual variability of herring^52^.

### Empirical dynamic modeling and convergent cross mapping

To understand the dynamic coupling between spring and fall abundances of *C. finmarchicus* and detect potential trophic control by various invertebrate and fish predators, we utilized CCM within the EDM framework. EDM is a set of time-series analysis methods based on nonparametric function approximation and state-space reconstruction (i.e., time-delay embedding) to analyze nonlinear dynamical systems. The theoretical foundation of EDM is Takens’ Theorem, which states that a multidimensional dynamical system can form a manifold in state space, and that time-delay embedding of a single time series can reconstruct a shadow version of the original manifold while preserving the system’s essential mathematical properties^53^. In other words, if variables are interacting within a time-invariant dynamical system, the time lags of a single one of those variables encapsulates information about the others, enabling recovery of the most important topological characteristics of the latent manifold^54^. CCM is a technique within this framework that identifies directional (and potentially causal) relationships between variables^10^. As an example of CCM, consider two variables X (e.g., zooplankton) and Y (e.g., fish predator). If X and Y are interacting in the same dynamical system such that X is driving Y (i.e., X → Y), then Y will inherently contain information about X. To quantify this impact, we can “cross-map” between the variables by using time lags of the effect (Y) time series to estimate how well it predicts the states of the driver (X) time series^55^. If X and Y are indeed dynamically coupled, the cross-mapping skill between these variables will increase as we increase the amount of data (or library size), since more data improves the resolution of the reconstructed shadow manifold. Thus, the CCM algorithm provides a more robust approach than mere correlation for inferring trophic interactions in complex ecological systems^54^.

In this analysis, we used seasonally-averaged time series of *C. finmarchicus* and potential predators to test the bottom-up effect (i.e., spring *C. finmarchicus* abundance → spring-summer predator abundance) and the top-down effect (i.e., spring-summer predator abundance → fall *C. finmarchicus* abundance). Since CCM requires evenly spaced temporal data, we used locally estimated scatterplot smoothing (LOESS) with a span of 0.5 to fill gaps for years in which no data was collected for that season. At most 11% of the data points were interpolated for the MARMAP/EcoMon data, and approximately 26% of the data were interpolated for herring abundance from the trawl survey. We normalized all data to zero mean and variance one so that the data were on the same scale, and we used simplex projection within the EDM framework to determine the best embedding dimension for the manifold reconstruction of the effect variable^55^. To assess significance and estimate a *p* value, we compared the final cross-mapping skill to the null distribution composed of the cross-mapping skill from 500 randomized seasonal surrogate time series. Each surrogate was generated to preserve the mean cyclical trend by computing the average signal over a specified period of 12 years and randomizing the residuals^10,56^. We applied this CCM procedure on data from Wilkinson and Jordan Basins (Figs. 2 and 4), and repeated the same approach for data from Stellwagen Bank (Supplementary Information: Figure S7). All CCM analyses were performed using the “rEDM” package in the R programming language (v.0.7.5).

### Boosted regression trees

We utilized boosted regression trees (BRTs) to assess which potential predators have the greatest predictive influence on *C. finmarchicus.* BRTs are a flexible, non-parametric statistical modeling technique that combine traditional regression trees with a boosting method, iteratively partitioning the data through recursive binary splitting. This algorithm results in an additive regression model that quantifies the influence of multiple predictors on a response variable based on the number of times each variable is chosen for splitting. This allows for a ranked assessment of the relative predictive influence of each variable on the response^57^. BRTs offer a data-driven assessment of the relative importance of multiple predictors, allowing us to identify which predators could have the strongest association with *C. finmarchicus* abundance. For this study, we excluded environmental covariates such as temperature or salinity, as our primary goal was to isolate and rank the relative predictive influence of potential predators on *C. finmarchicus* abundance, independent of abiotic environmental drivers. Therefore, we regressed both spring and fall *C. finmarchicus* interannual abundances against spring-summer abundances of five potential predators within the same year to assess the relative bottom-up effect of *C. finmarchicus* on potential predators (spring *C. finmarchicus* abundance as the response variable) and the top-down effect of each potential predator on *C. finmarchicus* (fall *C. finmarchicus* abundance as the response variable). We optimized our BRT model parameters, including the learning rate, tree complexity, and the optimal number of trees, using k-fold cross-validation following best practices for ecological data^57^. All BRT analyses were performed using the R package “gbm” (v.2.1.9).

## Supplementary Information

Below is the link to the electronic supplementary material.

Supplementary Material 1

## Data Availability

Plankton abundance data were collected by the MARMAP/EcoMon survey program, publicly available at the NOAA National Centers for Environmental Information site (https://www.ncei.noaa.gov/archive/accession/0187513—see reference^58^). Herring abundance data is publicly available at https://www.fisheries.noaa.gov/inport/item/22557 (see reference^59^). R scripts used to conduct the statistical analyses are available at https://github.com/iahonda/Trophic-Controls.git.
