# Supplementary material for: Seasonal trophic controls drive population variability in a foundational marine copepod
Source: Sci Rep. 2025 Oct 15;15:36018. doi: 10.1038/s41598-025-19919-2 (PMC12528431; doi:10.1038/s41598-025-19919-2)
Supplement: Supplementary file 1 — Supplementary Material 1 [file 41598_2025_19919_MOESM1_ESM.pdf]

Article Submitted to *Scientific Reports*

Seasonal Trophic Controls Drive Population Variability in a Foundational Marine Copepod

SUPPORTING INFORMATION

Authors:

Isabel A. Honda[1,2*], Lucas P. Medeiros[1], Cameron R.S. Thompson[1,3], Gregory L. Britten[1,4], Jeffrey A. Runge[5], Rubao Ji[1]

Affiliations:

[1]Biology Department, Woods Hole Oceanographic Institution, Woods Hole, MA

[2]Department of Civil and Environmental Engineering, Massachusetts Institute of Technology, Cambridge, MA

[3]Northeastern Regional Association of Coastal Ocean Observing Systems, Portsmouth, NH

[4]Department of Earth, Atmospheric, and Planetary Sciences, Massachusetts Institute of Technology, Cambridge, MA

[5]Darling Marine Center School of Marine Sciences, University of Maine, Walpole, ME

*Corresponding author: ihonda@mit.edu

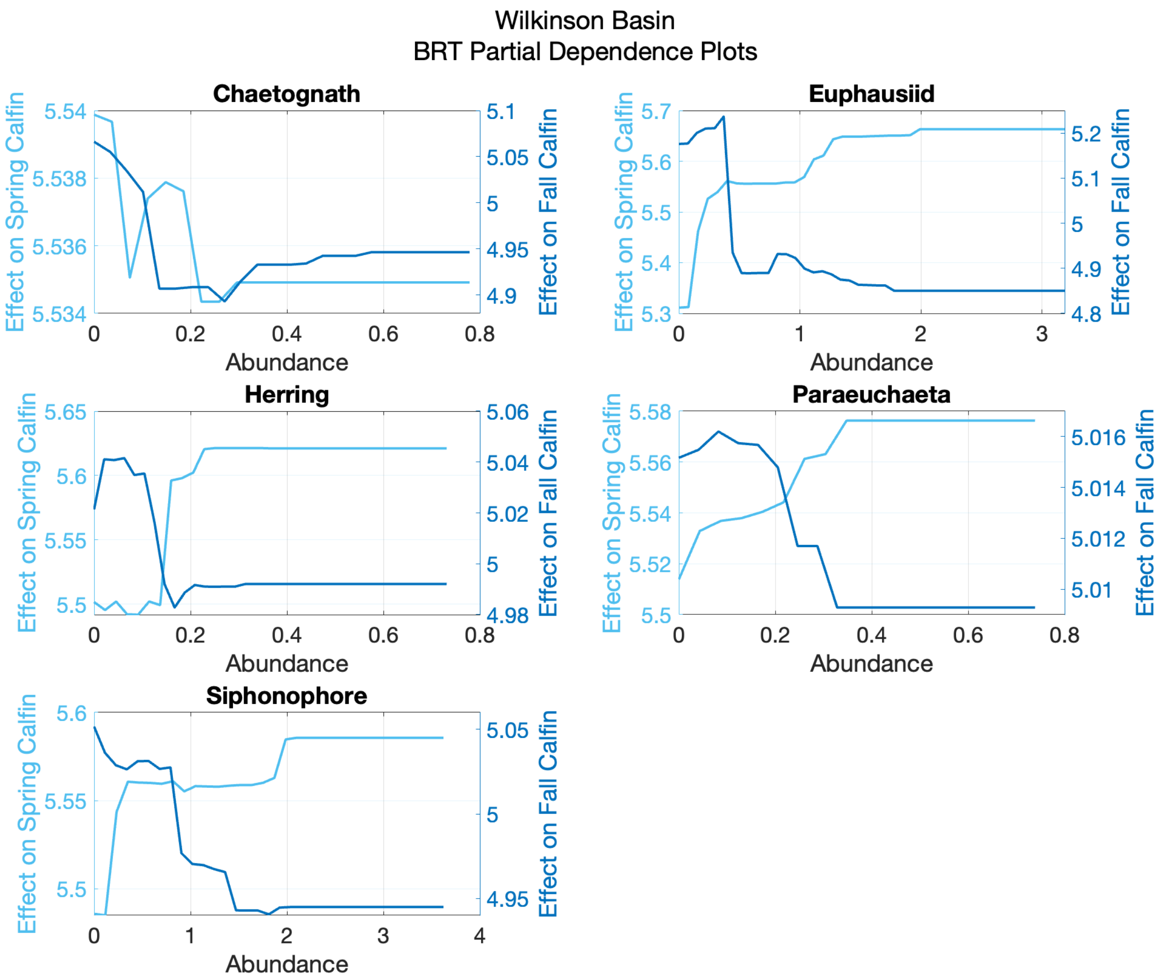

Figure S1: Partial dependence plots showing the average marginal effect of each predictor from the Boosted Regression Tree (BRT) for five potential predators of *C. finmarchicus* (Calfin) in Wilkinson Basin.

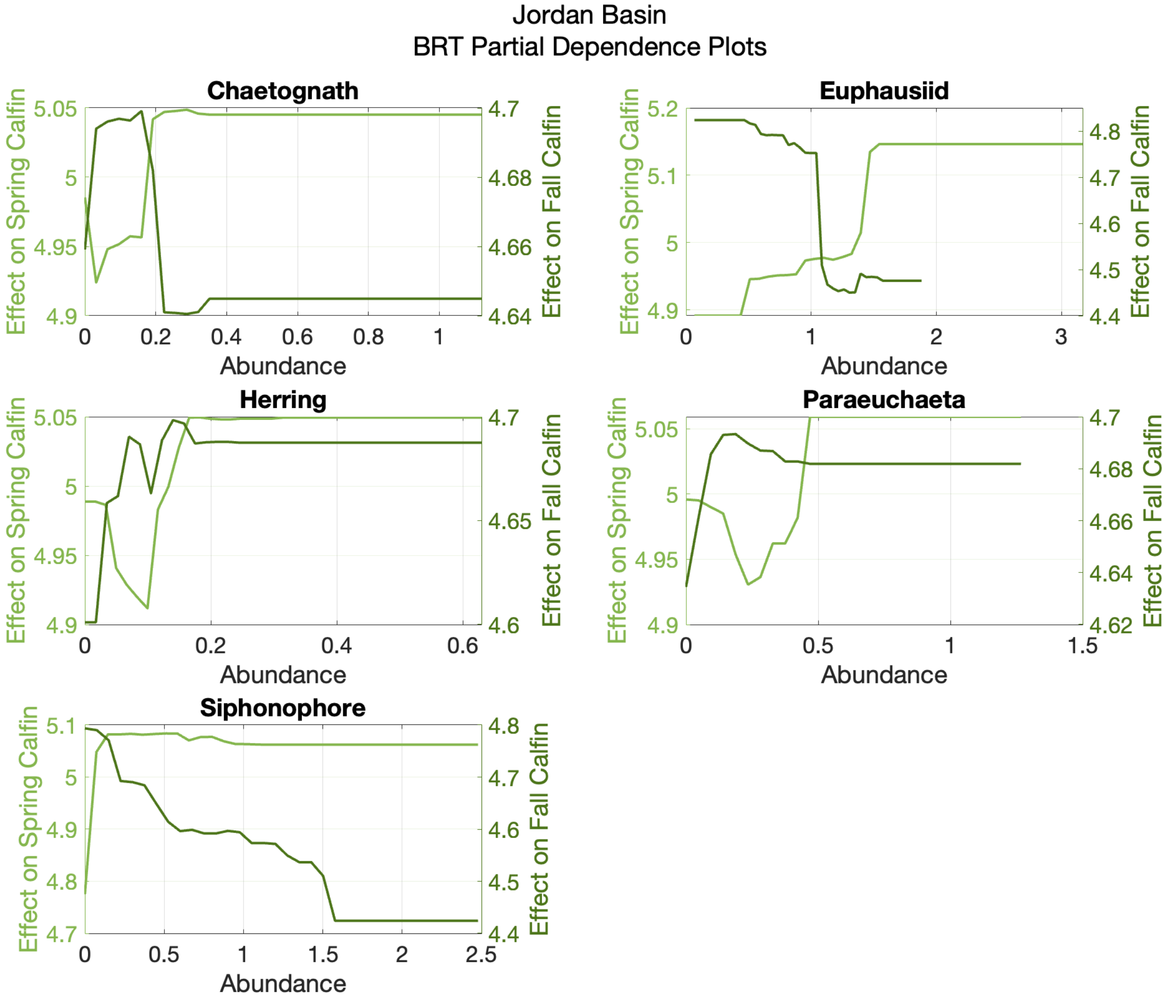

Figure S2: Partial dependence plots showing the average marginal effect of each predictor from the Boosted Regression Tree (BRT) for five potential predators of *C. finmarchicus* (Calfin) in Jordan Basin.

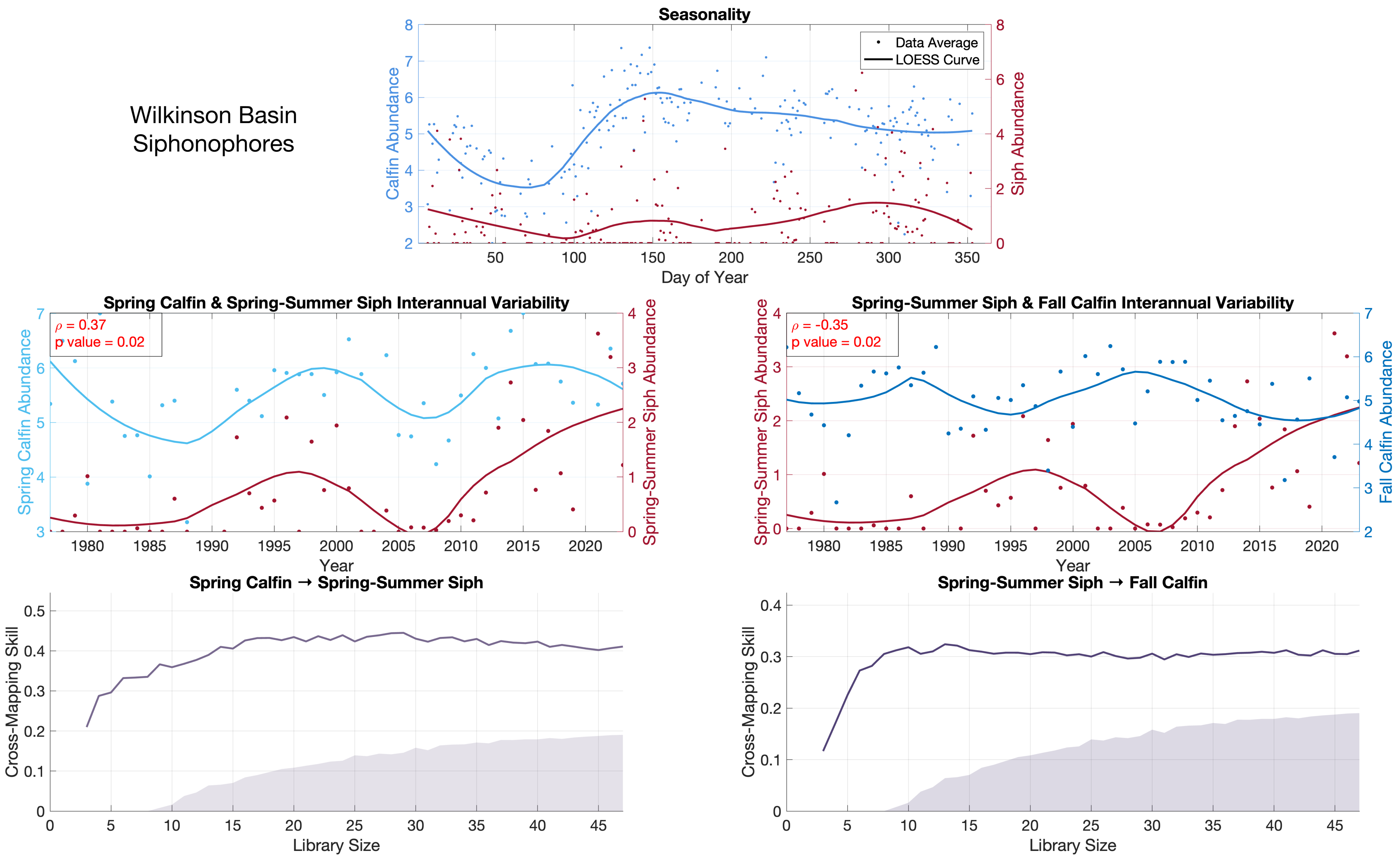

Figure S3: Seasonality, interannual variability, and CCM results analyzing Spring-Summer siphonophores (Siph) and Spring/Fall *C. finmarchicus* (Calfin) abundances in Wilkinson Basin. Points represent day of year or annual seasonal means; solid lines show LOESS-smoothed trends. Abundance values are in units of ln(abundance m^{-3} + 1).

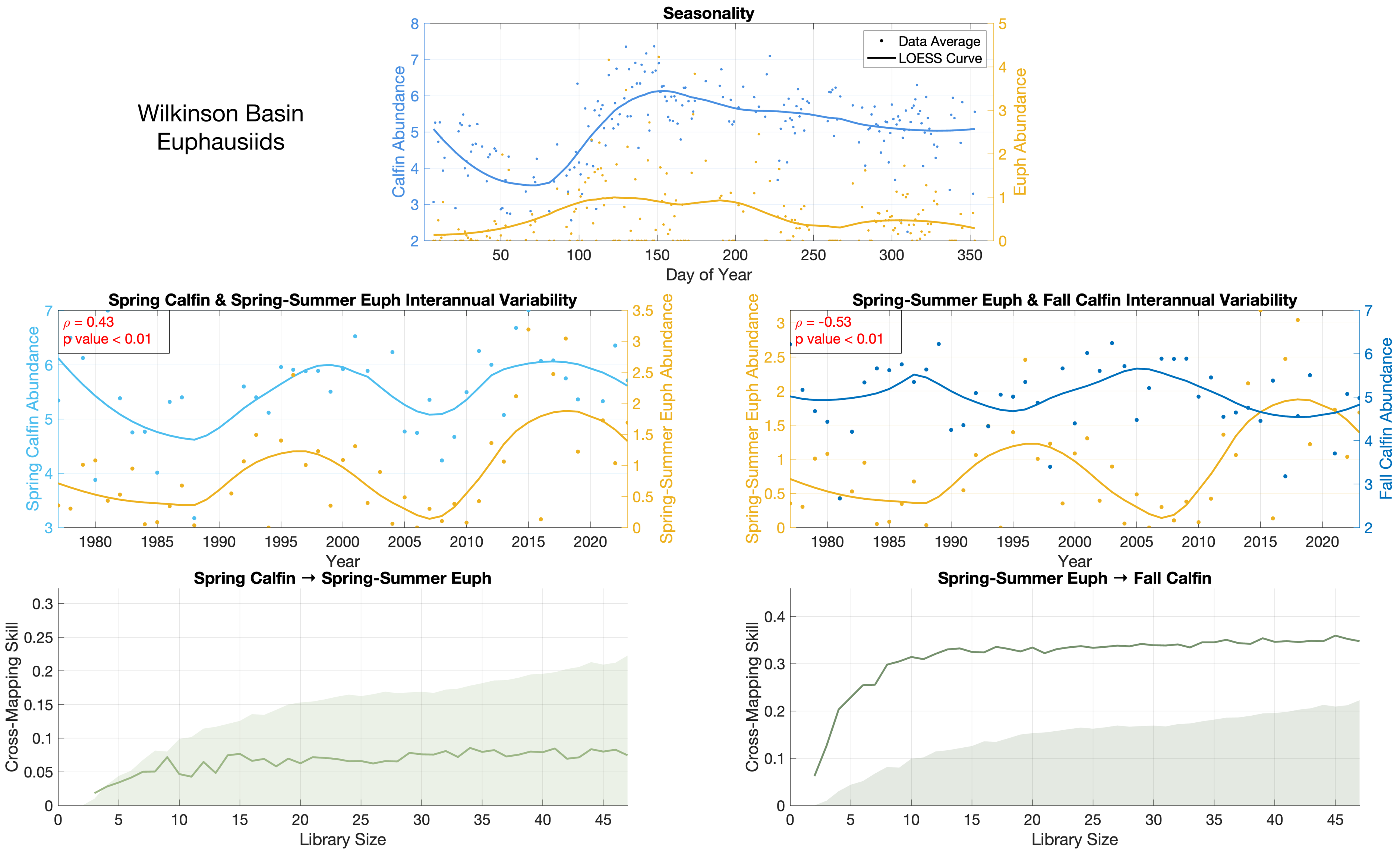

Figure S4: Seasonality, interannual variability, and CCM results analyzing Spring-Summer euphausiids (Euph) and Spring/Fall *C. finmarchicus* (Calfin) abundances in Wilkinson Basin. Points represent day of year or annual seasonal means; solid lines show LOESS-smoothed trends. Abundance values are in units of ln(abundance m^{-3} + 1).

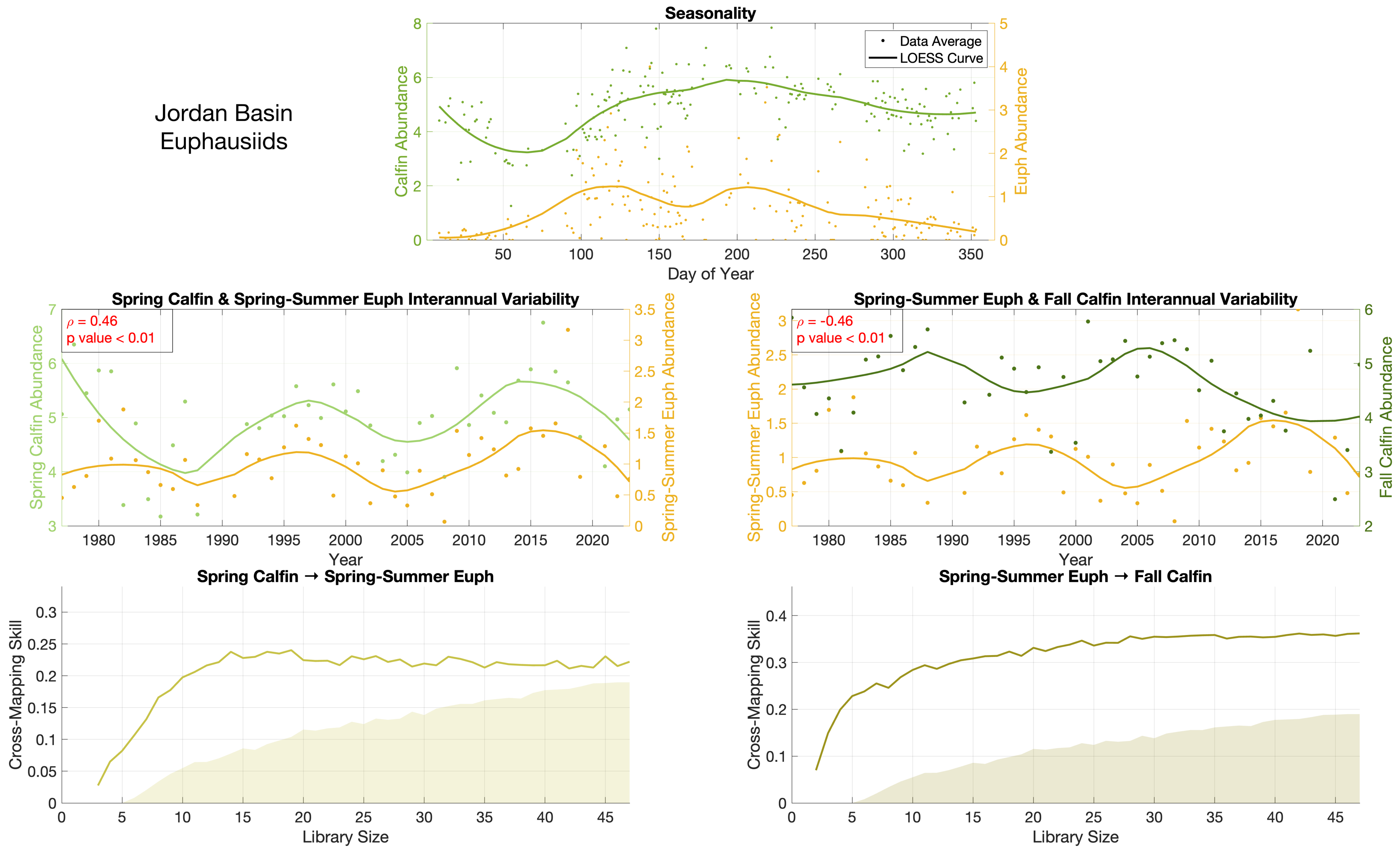

Figure S5: Seasonality, interannual variability, and CCM results analyzing Spring-Summer euphausiids (Euph) and Spring/Fall *C. finmarchicus* (Calfin) abundances in Jordan Basin. Points represent day of year or annual seasonal means; solid lines show LOESS-smoothed trends. Abundance values are in units of ln(abundance m^{-3} + 1).

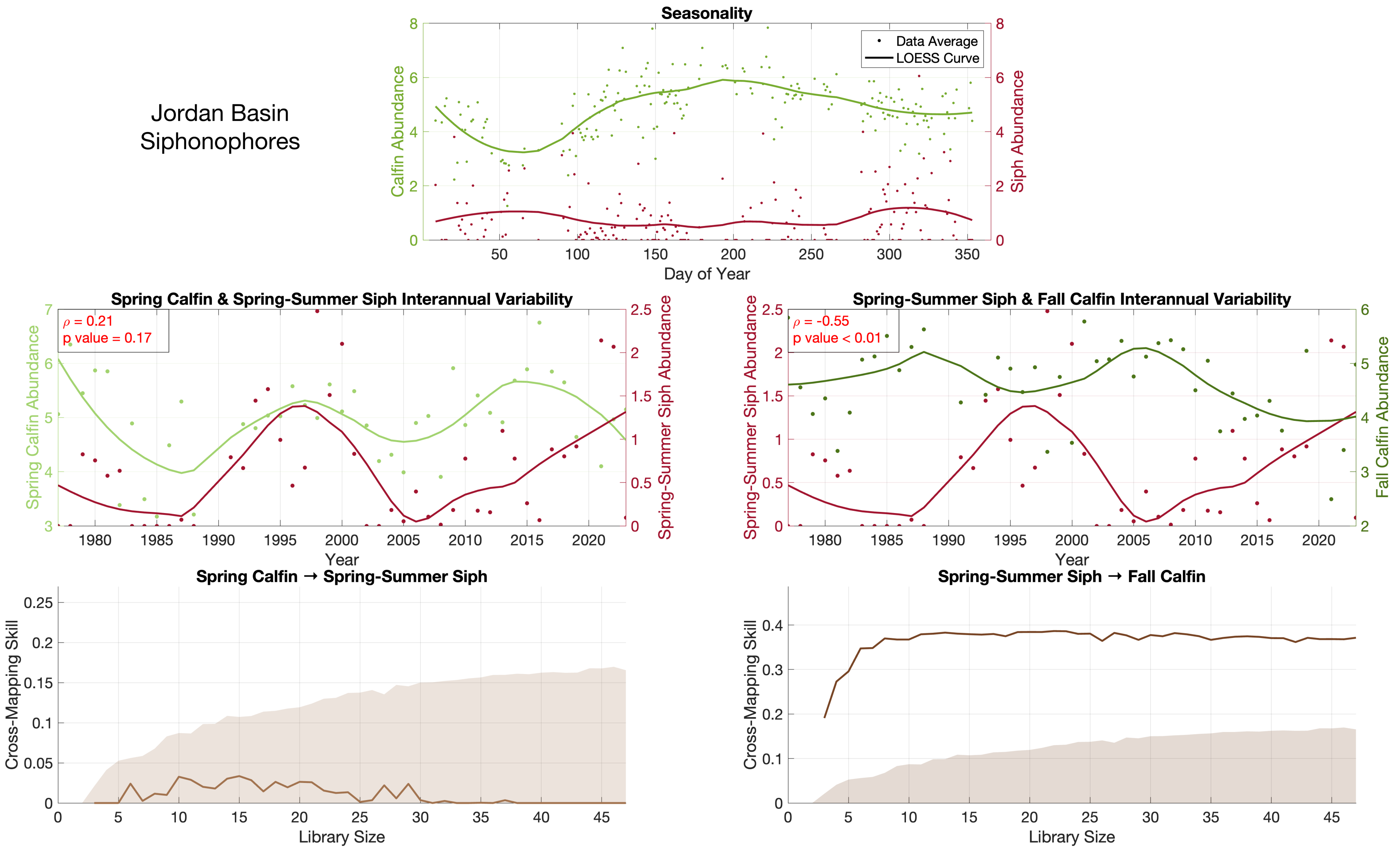

Figure S6: Seasonality, interannual variability, and CCM results analyzing Spring-Summer siphonophores (Siph) and Spring/Fall *C. finmarchicus* (Calfin) abundances in Jordan Basin. Points represent day of year or annual seasonal means; solid lines show LOESS-smoothed trends. Abundance values are in units of ln(abundance m^{-3} + 1).

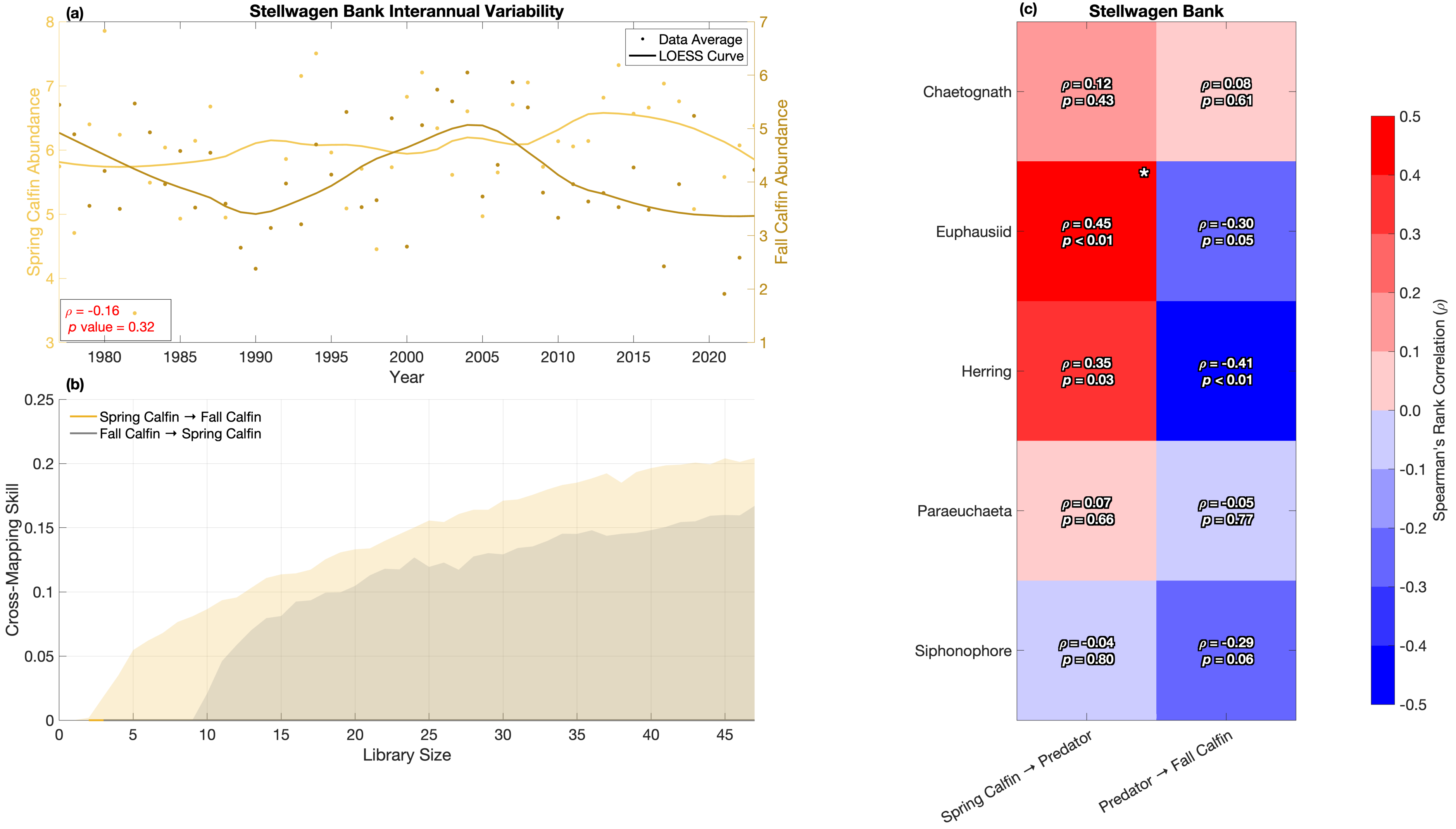

Figure S7: Spring → Fall CCM results for *C. finmarchicus* (Calfin) populations (a, b) and predator correlations/CCM results (c) on Stellwagen Bank. Points represent annual seasonal means; solid lines show LOESS-smoothed trends. Abundance values are in units of $\ln(\text{abundance m}^{-3} + 1)$.

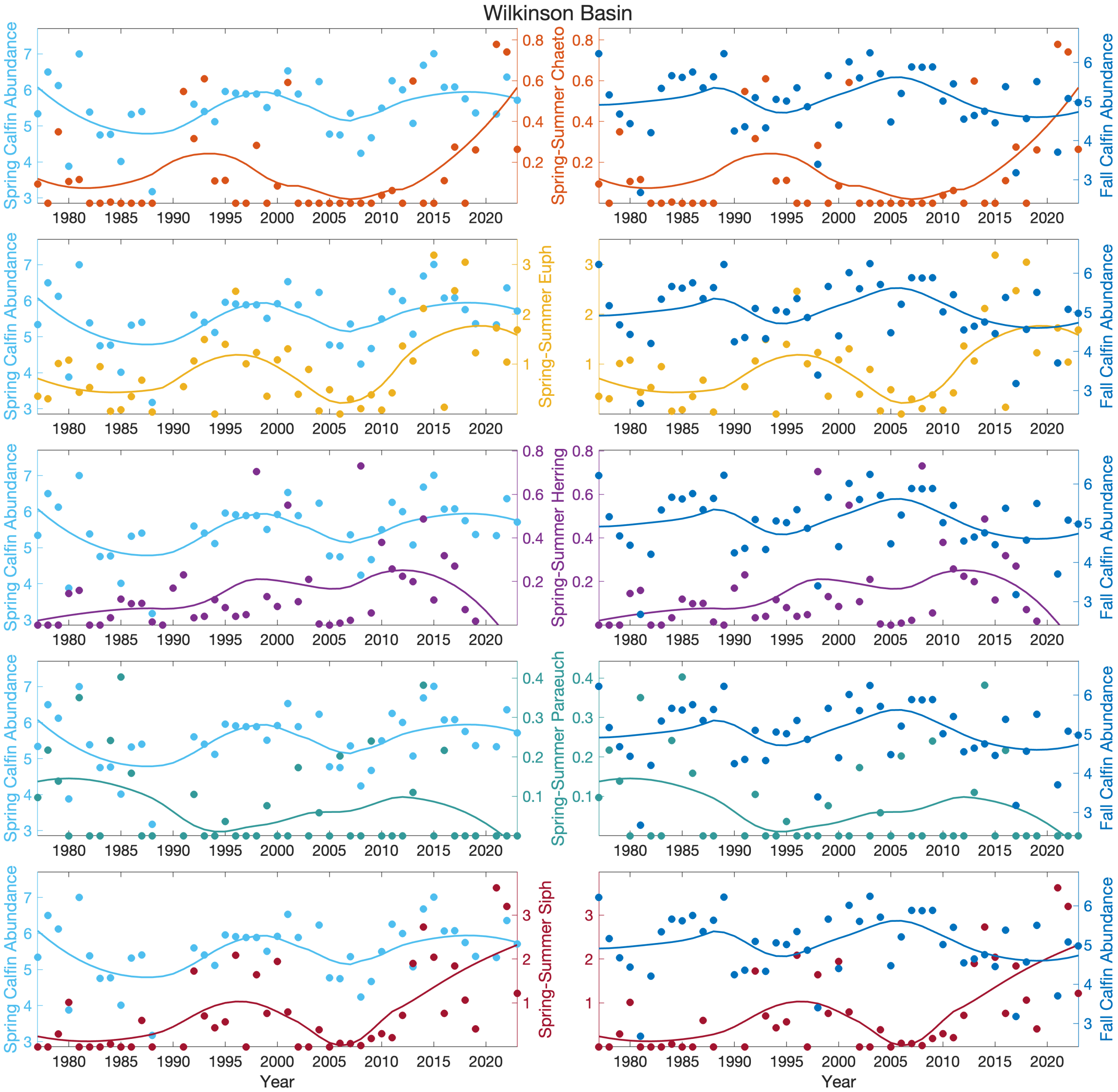

Figure S8: Interannual variability in seasonal abundance of five potential predators of *Calanus finmarchicus* (Calfin) in Wilkinson Basin. Each row corresponds to a different predator group—chaetognaths (Chaeto), euphausiids (Euph), Atlantic herring (Herring), *Paraeuchaeta* spp. (Paraeuch), and siphonophores (Siph)—with seasonal predator abundance plotted against Calfin abundance. Left column panels show predator abundance compared to Spring Calfin; right column panels show the same predator groups compared to Fall Calfin. Points represent annual season means; solid lines show LOESS-smoothed trends for better visualization. Y-values are log-transformed (in units of ln[abundance m^{-3} + 1] and ln[kg/ha + 1] for herring) and averaged across years within each season.

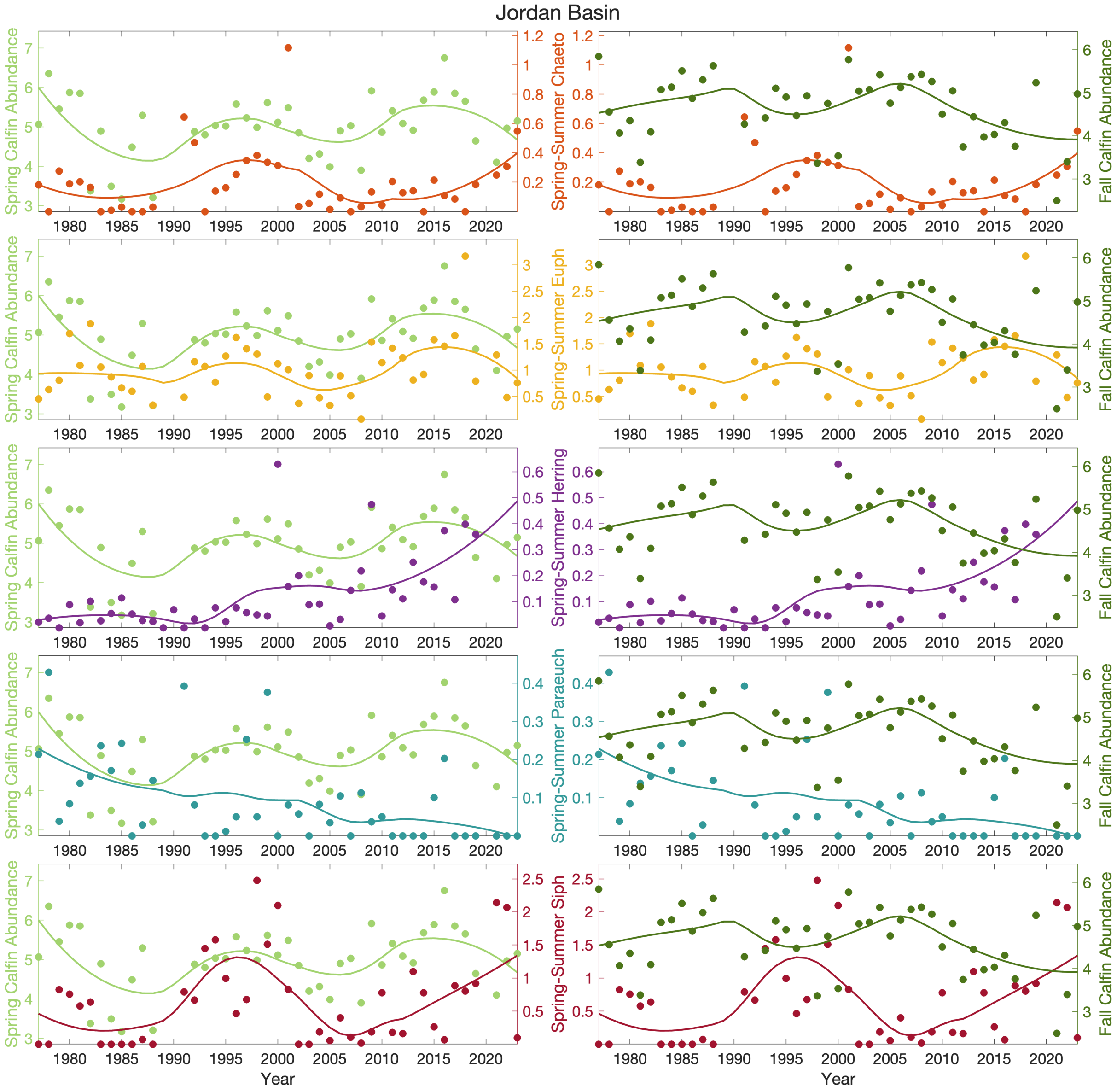

Figure S9: Interannual variability in seasonal abundance of five potential predators of *C. finmarchicus* (Calfin) in Jordan Basin. Each row corresponds to a different predator group—chaetognaths (Chaeto), euphausiids (Euph), Atlantic herring (Herring), *Paraeuchaeta* spp. (Paraeuch), and siphonophores (Siph)—with seasonal predator abundance plotted against Calfin abundance. Left column panels show predator abundance compared to Spring Calfin; right column panels show the same predator groups compared to Fall Calfin. Points represent annual season means; solid lines show LOESS-smoothed trends for better visualization. Y-values are log-transformed (in units of ln[abundance $m^{-3} + 1$] for invertebrates and ln[kg/ha + 1] for herring) and averaged across years within each season.
